# Polymerization of Cyclic Esters Initiated by Carnitine and Tin (II) Octoate

**DOI:** 10.3390/molecules14020621

**Published:** 2009-02-04

**Authors:** Marcin Sobczak, Waclaw Kolodziejski

**Affiliations:** Department of Inorganic and Analytical Chemistry, Faculty of Pharmacy, Medical University of Warsaw, ul. Banacha 1, 02-097 Warsaw, Poland; E-mail: waclaw@farm.amwaw.edu.pl (W. K.)

**Keywords:** Carnitine, Biomaterials, Polycaprolactone, Polylactide, Ring-opening polymerization

## Abstract

Low-molecular weight poly(ε-caprolactone), polylactides and copolymers of ε−caprolactone and lactides were obtained by the polymerization of cyclic esters in the presence of a carnitine/SnOct_2_ system. Their structures were proven by means of MALDI−TOF, IR and NMR studies. Effects of temperature, reaction time and carnitine dosage on the polymerization process were examined.

## Introduction

Pharmacy and medicine are among the most important application fields of polymers. Polymers are used in production of prosthetic and dental materials, artificial organs, sutures and disposable hygiene products, active macromolecular pharmaceutical substances, blood substitutes, auxiliary materials and excipients, macromolecular prodrugs, polymeric drug delivery systems, therapeutic systems, etc. The polymeric prodrugs, drug delivery systems and therapeutic systems exhibit unique pharmacokinetics, body distribution and pharmacological efficacy [1,2,3,4,5,6,7,8,9,10,11,12,13,14,15,16]. 

Aliphatic polyesters are typical biomaterials, commonly used in medicine and pharmacy because of their good biocompatibility and lack of toxicity. The majority of the products are composed of homo- and copolymers of lactides (LA, LLA) and ε-caprolactone (CL) [1,2,14,15]. Aliphatic polyesters are usually prepared by ring-opening polymerization (ROP) of the relevant cyclic monomers (e.g. d,l-, l,l-lactide, ε−caprolactone; abbreviations: LA, LLA, CL, respectively). PLA, PLLA and PCL have been successfully synthesized by ring opening polymerization in the presence of cationic or anionic initiators, as well as coordinating and enzymatic catalysts [16,17,18,19,20,21,22,23,24,25,26,27,28,29,30,31,32,33,34,35,36,37,38]. The tin octoate (SnOct_2_) is probably the most often used catalyst in the polymerization of cyclic esters. 

l-Carnitine (L-CA) is a hydrophilic amino acid derivative, naturally occurring in human cells. The compound is biosynthesized endogenously in the kidneys and liver from lysine and methionine, but it can also be delivered with red meat and dairy products of the diet. l−Carnitine plays an essential role in the transfer of long-chain fatty acids into mitochondria for beta-oxidation. Furthermore, L-carnitine binds acyl residues and helps in their elimination, decreasing the number of acyl residues conjugated with coenzyme A (CoA) and increasing the ratio between free and acylated CoA. Carnitine deficiency is a pathologic metabolic state in which carnitine concentrations in plasma and tissues are lower than the levels required for normal functioning of the organism [39].

Recently, we found that natural amino acids are satisfactory initiators for ROP of cyclic esters [34]. In the present paper, we describe a new effective synthesis of low−molecular weight aliphatic polyesters. It involves the ring opening polymerization of d,l-, l,l-lactide, ε-caprolactone in the presence of a L-carnitine/SnOct_2_ system. We believe that thus obtained polymers can be practically applied as effective drug delivery systems.

## Results and Discussion

**Scheme 1:**
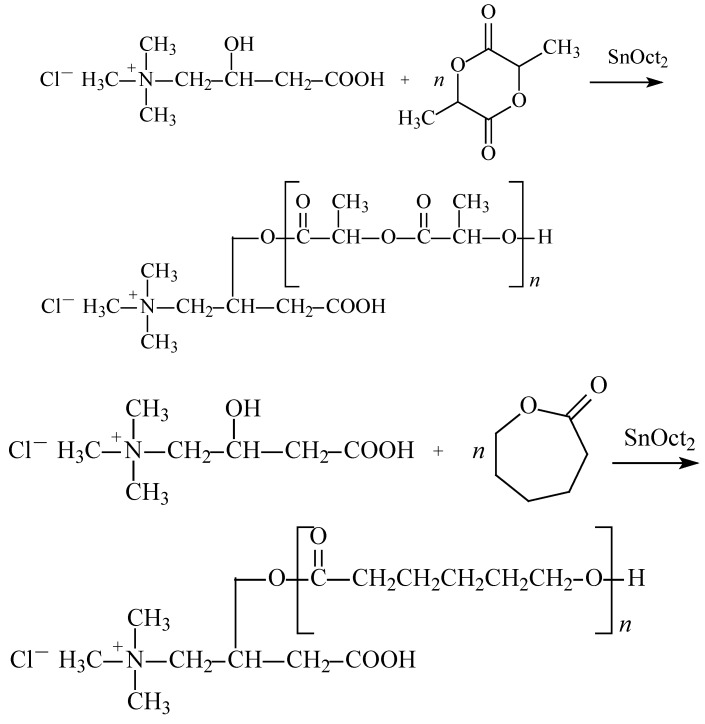
The synthesis scheme of oligoesters.

The homo- and copolymerization reactions of CL, LA and LLA were carried out in the presence of the CA/SnOct_2_ (2:1) system at 120-160°C. The molar ratio of CA to a given monomer was 1:25, 1:50 or 1:100. Reaction conditions, yields and average molecular mass values of polyesters are summarized in Table 1. A typical reaction scheme is presented in Scheme 1.

**Table 1:** Homo- and copolymerization of cyclic esters.

Symbol	M	M/CA	Time (h)	Temp. (°C)	Yield (%)	M_n _^th^ (Da)	M_n _^a^ (Da)	PD ^a^	M_n_^b^ (Da)	PD ^b^	M_n _^c^ (Da)	L_CL_^d^ (% mol)
**PCL-1**	CL	25:1	24	120	62	1767	-	-	1500	1.1	1900	-
**PCL-2**	CL	25:1	24	140	85	2423	2200	1.2	1800	1.1	2400	-
**PCL-3**	CL	50:1	24	140	71	4047	-	-	3800	1.2	3100	-
**PCL-4**	CL	50:1	24	160	93	5301	4800	1.1	5500	1.2	6100	-
**PCL-5**	CL	100:1	24	140	67	7638	-	-	6600	1.2	6300	-
**PCL-6**	CL	100:1	48	140	81	9234	-	-	8800	1.3	6500	-
**PCL-7 ^e^**	CL ^e^	50:1	72	160	33	1881	1400	1.1	1600	1.2	1200	-
**PLA-1**	LA	25:1	24	140	62	2232	-	-	1700	1.2	1600	-
**PLA-2**	LA	50:1	24	140	53	3816	-	-	3200	1.2	2700	-
**PLA-3**	LA	50:1	48	140	68	4896	3800	1.3	4200	1.2	5100	-
**PLA-4**	LA	100:1	24	120	30	4320	-	-	3800	1.3	4200	-
**PLA-5**	LA	100:1	24	140	36	5184	-	-	4600	1.2	3900	-
**PLA-6 ^e^**	LA ^e^	50:1	72	140	21	1512	-	-	1200	1.1	1000	-
**PLLA-1**	LLA	50:1	24	120	32	2304	2100	1.2	1900	1.1	2700	-
**PLLA-2**	LLA	50:1	24	140	57	4104	-	-	3600	1.1	3200	-
**PCLLA-1 **	CL/LA	25:25:1	24	140	51	3290	-	-	2800	1.2	-	56
**PCLLA-2 **	CL/LA	25:25:1	48	140	58	3741	-	-	3000	1.2	-	58

*Reaction conditions*: argon atmosphere, CA/SnOct_2_ (2:1); M – monomer, CA – carnitine, LA – *rac*-lactide, LLA - l-lactide, CL - ε-caprolactone, L_CL_ - ε-caprolactone units content in copolymer chain; M_n_^th^ – theoretical molecular weights, M_n_^th^ = [M]/[CA] · M_mon_ · conversion (%) (for homopolymers), M_n_^th^ = ([M_1_]/[CA] · M_mon1_ + [M_2_]/[CA] · M _mon2_) · conversion (%) (for copolymers); ^a^ determined by MALDI-TOF; ^b^ determined by GPC; ^c^ determined by viscosity method; ^d^ determined by ^1^H-NMR: L_CL_^c^ (in CL/LA) = (signal intensity of the –C(O)C**H_2_**CH_2_CH_2_CH_2_CH_2_O-/ signal intensity of the −OC(O)C**H**(CH_3_)O-)·100; ^e^ reaction without SnOct_2_

**Figure 1:**
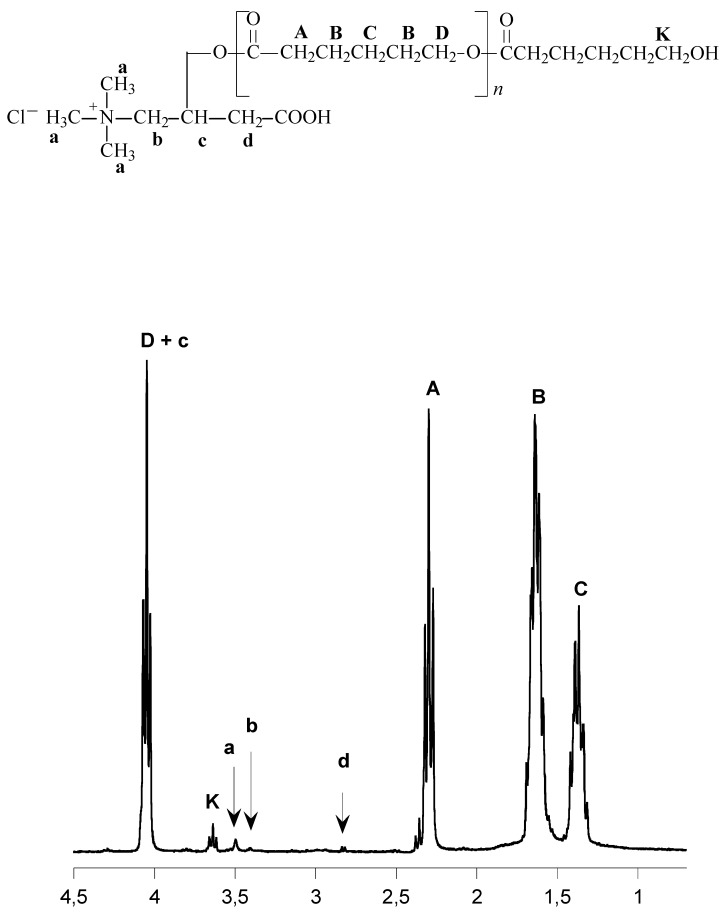
^1^H-NMR spectra of the CL homopolymer produced in the presence of carnitine and SnOct_2 _(PCL-4).

**Figure 2:**
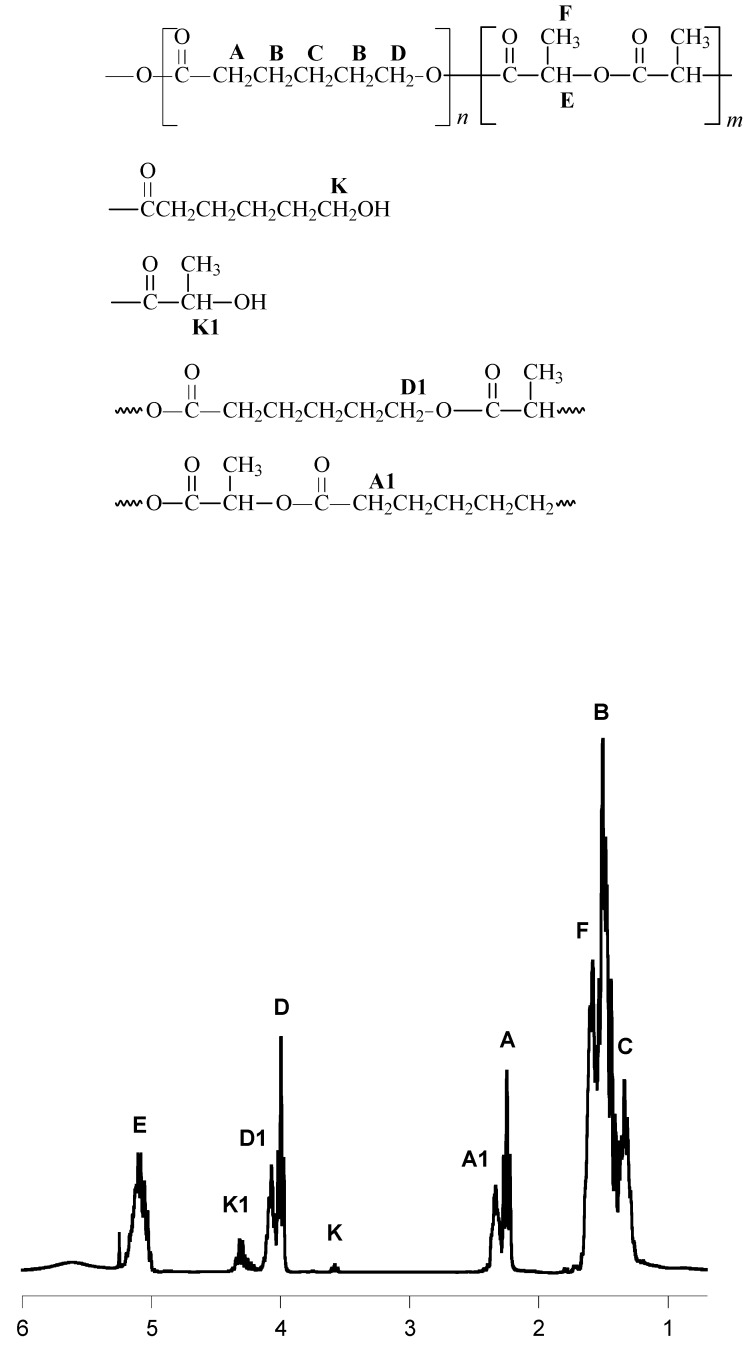
^1^H-NMR spectra of the CL/LA copolymer produced in the presence of carnitine and SnOct_2_ (PCLLA-2).

The chemical structures of the obtained polymers were confirmed by ^13^C, ^1^H-NMR and IR studies (Figure 1 and Figure 2, Table 2 and Table 3). 

**Table 2:** ^1^H- and ^13^C-NMR structural assignments of the synthesized polyesters (spectrum recorded in chloroform at room temperature).

Chemical shift in ppm	Structural assignments
*poly(ε-caprolactone)*	
4.01	(2H, t, -CH_2_C**H**_2_OC(O)-)
3.70	(2H, t, -CH_2_C**H**_2_OH, end group)
2.24	(2H, t, -CH_2_C**H**_2_COO-)
1.58	(4H, m, -C**H**_2_CH_2_COO-)
1.33	(2H, m, -CH_2_CH_2_C**H**_2_CH_2_CH_2_-)
173.1	(-**C**(O)O-)
63.7	(-CH_2_**C**H_2_OC(O)-)
33.6	(-CH_2_**C**H_2_COO-)
27.9	(-**C**H_2_CH_2_OC(O)-)
25.1	(-**C**H_2_CH_2_COO-)
24.1	(-CH_2_CH_2_**C**H_2_CH_2_CH_2_-)
*polylactides*	
5.17	(1H, q, -C**H**(CH_3_)-)
4.36	(1H, q, -C**H**(CH_3_)OH, end group)
1.58	(3H, d, -C**H**_3_)
169.80	(-**C**(O)O-)
69.2	(-**C**H(CH_3_)-)
16.8	(-**C**H_3_)
*ε-caprolactone and lactide copolymers*	
5.15	1H, q, -C**H**(CH_3_)-
4.27	1H, q, -C**H**(CH_3_)OH, end group
4.11	2H, t, -CH_2_C**H**_2_OC(O)-LA
4.03	2H, t, -CH_2_C**H**_2_OC(O)-
3.67	2H, t, -CH_2_C**H**_2_OH, end group
2.37	2H, t, -CH_2_CH_2_CH_2_C**H**_2_COO-LA
2.29	2H, t, -CH_2_C**H**_2_COO-
1.63	4H, m, −C**H**_2_CH_2_COO−
1.59	3H, d, -C**H**_3_
1.34	2H, m, -CH_2_CH_2_C**H**_2_CH_2_CH_2_-

**Table 3:** Main absorption bands of the synthesized polyestres (spectrum recorded from a KBr pellet).

Wave number in cm ^-1^	Group and band
*Poly(ε-caprolactone)* 2943 (υ_as_CH_2_), 2862 (υ_s_CH_2_), 1721 (υC=O), 1291 (C-O and C-C) 1240 (υ_as_COC), 1190 (υOC-O), 1170 (υ_s_COC), 1157 (C-O and C-C)	
*Polylactides* 2997 (υ_as_CH_3_), 2947 (υ_s_CH_3_), 2882 (υCH), 1760 (υC=O), 1452 (δ_as_CH_3_), 1348-1388 (δ_s_CH_3_), 1368−1360 (δ_1_CH+δ_s_CH_3_), 1315-1300 (δ_2_CH), 1270 (δCH + υCOC), 1215-1185 (υ_as_COC + r_as_CH_3_), 1130 (r_as_CH_3_), 1100-1,090 (υ_s_COC), 1045 (υC-CH_3_), 960-950 (rCH_3_ + υCC), 875-860 (υC-COO), 760-740 (δC=0), 715-695 (γC=O), 515 (δ_1_C-CH_3_ + δCCO), 415 (δCCO), 350 (δ_2_C-CH_3_ + δCOC), 300-295 (δCOC + δ_2_C-CH_3_), 240 (τCC)	

Insertion of the carnitine fragment into the polymer chain was confirmed by the proton NMR spectral analysis. The peaks at 2.83 (-C**H_2_**COOH), 3.51 ((C**H_3_**)_3_N^+^-) and 3.43 (−C**H_2_**N^+^-) ppm were observed in all products obtained by homo- and copolymerization of CL, LA and LLA in the presence the carnitine/SnOct_2_ system. 

Composition of the CL and LA (PCLLA) copolymers was deduced from the ^1^H-NMR spectra. The CL content in the copolymer of CL and LA exceeded the CL feed ratio for PCLLA (amounts to 56-58 mol %). Probably, CL is the most active co-monomer in this reaction. 

**Figure 3:**
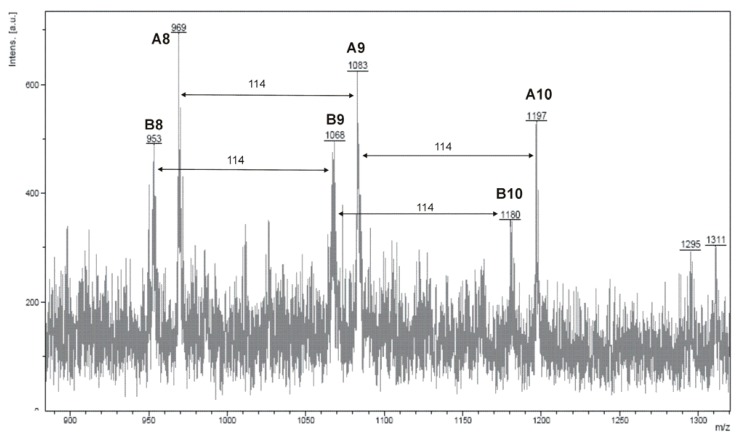
MALDI TOF spectra of the product of CL polymerization in the presence carnitine and SnOct_2_. A10(B10) = -[C(O)CH(CH_3_)O]_10_-, etc (PCL-2).

The MALDI-TOF spectra of PCL contain double peaks, each component corresponding to a separate spectrum series. The most prominent series of peaks is characterized by a mass increment of 114 Da, which is equal to the mass of the repeating unit in the poly(ε-caprolactone) (Figure 3). It is assigned to PCL terminated with a hydroxyl group (residual mass: 57 Da, K^+^ adduct) (A). The second series of peaks also corresponded to poly(ε-caprolactone), terminated with a hydroxyl group (residual mass: 40 Da, Na^+^ adduct) (B). 

In the MALDI-TOF spectra of PLA there are also two series of peaks. The main series corresponds to PLA molecules, terminated with a hydroxyl group (residual mass: 41 Da, Na^+^ adduct), and the second series of smaller peaks corresponds also to PLA terminated with a hydroxyl group (residual mass: 57 Da, K^+^ adduct). In the MALDI-TOF spectrum of PLA both populations of chains of even and odd number of lactic acid m.u. can be observed. The odd number of acid m.u. shows that under the reaction conditions the polymer chain undergoes intermolecular transesterification (leading to an exchange of segments), which is a typical phenomenon for the polymerization of lactides [18].

The molecular mass of PCL, PLA and PLLA is dependent on the monomer/carnitine molar ratio (Table 1). The influence of the monomer/carnitine feed ratio on the molecular weight of polyesters was studies at three levels (25:1, 50:1, 100:1). As shown in Table 1, the PCL products were obtained with M_n_ (from GPC) of 1800, 3800 and 6600 Da for PCL-2, PCL-3 and PCL-5, respectively. For PLA, M_n_ (from GPC) amounts to 1700, 3200 and 4600 Da for PLA-1, PLA-2 and PLA-5, respectively. It was found that the molar mass of the polyesters increased with the monomer/carnitine feed ratios. On the other hand, according to M_n_ of polyesters, the PCL and PLA conversion had tendency to decrease with the increasing monomer/carnitine feed ratio. For PCL−2, PCL-3, PCL-5, PLA-1, PLA-2 and PLA-5 the corresponding monomer conversion values were 85%, 71%, 67%, 62%, 53% and 36%, respectively. The reaction yield was determined by the weight method. The homo- and copolymerization reactions of CL, LA and LLA were repeated twice for each combination. The results were in good agreement with one another (reproducibility of them was about 5-10%). Both, the conversion and molecular mass of the polymers increased, when the reaction temperature was raised from 120 to 160ºC. 

The average molecular mass values of PCL determined by the MALDI-TOF method are in the 1400–4800 Da range and according to the viscosity measurement are in the 1200-6500 Da range. For polylactides, the M_n_ values determined from MALDI-TOF and the viscosity measurements are 2100‒3800 and 1000-5100 Da, respectively. M_n_ determined from GPC for CL oligomers is in the range of 1500–8800 Da (polydispersity indexes 1.1–1.3). For polylactides the M_n_ values are 1200–4600 Da and M_w_/M_n_ = 1.1−1.3.

The molecular mass values averaged over those of the obtained polymers were roughly in agreement with the theoretical molecular weights calculated from the feed ratio of the monomer to carnitine as well as the number average molecular mass determined from MALDI-TOF and GPC. 

Finally, it should be mentioned, that the carnitine/SnOct_2_ system was quite effective in the polymerization of ε-caprolactone, l-lactide and *rac*-lactide. The yield of PCL was in the range of 62−93 %, and for PDLA in the range of 30-68 %. Relevant kinetic and mechanistic studies are underway. They will be presented in the next paper.

## Experimental Section

### Materials

ε-Caprolactone (2-Oxepanone, 99%, CL) was purchased from Aldrich. Before use, it was dried and distilled over CaH_2_ at reduced pressure. 3,6-Dimethyl-1,4-dioxane-2,5-dione, (*rac*-lactide, 98%, LA) and (3*S*)-*cis*-3,6-Dimethyl-1,4-dioxane-2,5-dione (l-lactide, 98%, LLA) (Aldrich) were crystallized from a mixture of dry toluene with hexane and dried at room temperature under vacuum. l-Carnitine hydrochloride (98%, CA, Aldrich) was dried at room temperature under vacuum for 2h. Stannous octoate (tin (II) 2-ethylhexanoate, 95%, SnOct_2_, Aldrich) was used as received.

### Polymerization Procedure

Polymerization of homo- and copolymers of cyclic esters were carried out in the same way. Monomers (CL, LA, LLA) and CA were placed in 10 mL glass ampoules under an argon atmosphere. The reaction vessels were then left standing at the required temperature in a thermostated oil bath for the appropriate time (Table 1). When the reaction was complete, the cold product was dissolved in dichloromethane, the obtained solution was washed with methanol and dilute hydrochloric acid (5% aqueous solution) under vigorous stirring. The latter operation was repeated three times. The isolated powdery or oily polymer was dried in vacuum for 72 h. Purity of the isolated polymers was tested by ^1^H-NMR.

### Measurements

The polymerization products were characterized by means of ^1^H- and ^13^C-NMR (Varian 300 MHz), and FT-IR spectroscopy (Perkin-Elmer). The NMR spectra were recorded in CDCl_3_. The IR spectra were measured from KBr pellets. Relative molecular mass values and molecular mass distributions were determined using MALDI-TOF and gel permeation chromatography (GPC). The MALDI-TOF spectra were measured in the linear mode on a Kompact MALDI 4 Kratos analytical spectrometer using a nitrogen gas laser and 2−[(4−hydroxyphenyl)diazenyl] benzoic acid (HABA) as a matrix. Molecular mass values and molecular mass distributions of polymers were determined at 308 K on a Lab Alliance gel permeation chromatograph equipped with Jordi Gel DVB Mixed Bed (250 mm x 10 mm) columns and a refractive detector, using THF or chloroform as eluent (1 mL/min). The molecular mass scale was calibrated with polystyrene standards.

Polymer viscosity was measured in chloroform (at 25ºC) and *N,N*-dimethylformamide (at 30ºC) using an Ubbelohde viscometer. Polymer molecular mass values were calculated from the Mark-Houwink formula using the following equation constants: K= 2.21·10^−4^ ml/g and *α* = 0.77 (for PLA), K= 3.25·10^−4^ ml/g and *α* = 0.77 (for PLLA), K = 1.94·10^−4^ and *α* = 0.73 (for PCL) [34].

*Sample Availability:* Contact the authors.
